# Salidroside ameliorates Adriamycin nephropathy in mice by inhibiting β‐catenin activity

**DOI:** 10.1111/jcmm.14340

**Published:** 2019-04-16

**Authors:** Xinzhong Huang, Haiyan Xue, Jinyu Ma, Yunzhong Zhang, Jing Zhang, Yue Liu, Xiaogang Qin, Cheng Sun

**Affiliations:** ^1^ Department of Nephrology Affiliated Hospital of Nantong University Nantong China; ^2^ Key Laboratory for Neuroregeneration of Jiangsu Province and Ministry of Education Nantong University Nantong China; ^3^ School of Medicine Nantong University Nantong China; ^4^ Department of Nephrology Traditional Chinese Medicine Hospital of Tongzhou District Nantong China

**Keywords:** Adriamycin nephropathy, podocytes, salidroside, β‐catenin

## Abstract

Salidroside is a major phenylethanoid glycoside in *Rhodiola rosea* L., a traditional Chinese medicine, with multiple biological activities. It has been shown that salidroside possesses protective effects for alleviating diabetic renal dysfunction, contrast‐induced‐nephropathy and other kidney diseases. However, the involved molecular mechanism was still not understood well. Herein, we examined the protective effects of salidroside in mice with Adriamycin (ADR)‐induced nephropathy and the underlying molecular mechanism. The results showed that salidroside treatment ameliorates proteinuria; improves expressions of nephrin and podocin; and reduces kidney fibrosis and glomerulosclerosis induced by ADR. Mechanistically, ADR induces a robust accumulation of β‐catenin in the nucleus and stimulates its downstream target gene expression. The application of salidroside largely abolishes the nuclear translocation of β‐catenin and thus inhibits its activity. Furthermore, the activation of β‐catenin almost completely counteracts the protective roles of salidroside in ADR‐injured podocytes. Taken together, our data indicate that salidroside ameliorates proteinuria, renal fibrosis and podocyte injury in ADR nephropathy, which may rely on inhibition of β‐catenin signalling pathway.

## 1 INTRODUCTION

Proteinuria is not only an early and pivotal pathologic feature but also a known indicative sign for progressive glomerular disease.^1^, ^2^ Furthermore, it contributes to the development and progression of chronic kidney disease (CKD) and end‐stage renal failure.^3^, ^4^ There are three layers for composing glomerular filtration barrier: inner fenestrated endothelium, glomerular basement membrane and outer glomerular visceral epithelium. Increasing evidence suggests that podocyte dysfunction is a key causative factor for glomerular filtration impairment and proteinuria.^5^ Therefore, the primary goal of glomerular disease treatment was to reduce proteinuria in a short time to protect the kidney.^6^

Salidroside is a phenylpropanoid glycoside compound with various pharmacological activities, including anti‐oxidative stress, anti‐fatigue, anti‐inflammation, anti‐aging and anti‐diabetes.^7^, ^8^, ^9^, ^10^, ^11^, ^12^ Salidroside has been found in food and traditional Chinese medicine, including *Osmanthus fragrans* flower, *Olea europaea* L. fruit, *Striga asiatica*, *Cistanche deserticola* Ma and *Rhodiola rosea* L.^13^, ^14^ Current evidences indicate that salidroside has protective roles on early nephropathy in alloxan‐induced diabetic mice 15 and db/db mice.^16^ Notably, these studies were carried out in mouse models of diabetic nephropathy; therefore, the protective roles of salidroside against kidney dysfunction may rely on its euglycemic activity.^10^, ^17^ Thus, whether salidroside has direct beneficial effects on kidney function is still unknown.

Wnt/β‐catenin, a conserved signalling pathway, plays an essential role in development. As for the kidney, Wnt/β‐catenin regulates nephron formation during embryogenesis and involves in injury repair and pathogenesis of kidney diseases.^18^ Once binding to its receptors, Wnts initiate a series of downstream cascades and lead to β‐catenin dephosphorylation. As a consequence, the stability of β‐catenin becomes stronger and then it translocates into the nucleus, where β‐catenin interacts with T cell factor and/or lymphoid enhancer‐binding factor to induce its target gene expression.^19^ It has been shown that an early and transient up‐regulation of Wnt/β‐catenin after acute kidney injury facilitates tubular repair and kidney regeneration.^20^ However, chronic activation of this signalling seems to be detrimental, leading to development and progression of kidney diseases.^21^

In the present study, the animal model of ADR nephropathy, a widely recognized rodent model for proteinuric kidney diseases, was employed to examine the therapeutic mechanisms of salidroside. The current data showed that salidroside improves kidney structure and function in ADR nephropathy, and these beneficial roles are likely dependent upon the inhibition of β‐catenin signalling pathway.

## 2 METHODS

### 2.1 Chemicals and reagents

Salidroside (purity >98%; catalog no. 110818) was from National Institute for Food and Drug Control (Beijing, China). ADR (doxorubicin hydrochloride; purity >98%; catalog no. D1515) and the antibody anti‐α‐tubulin (catalog no. T9026) were provided from Sigma‐Aldrich (St. Louis, MO, USA). The antibodies anti‐nephrin (catalog no. ab216341), anti‐podocin (catalog no. ab50339), anti‐β‐catenin (catalog no. ab32572), anti‐fibronectin (catalog no. ab2413), anti‐collagenⅠ(catalog no. ab34710) and anti‐β‐Actin (catalog no. ab8226) were purchased from Abcam (Cambridge, MA, USA). The antibodies anti‐α‐SMA (smooth muscle actin) (catalog no. #19245), anti‐Lamin A/C (catalog no. #4777), anti‐MMP7 (matrix metalloproteinase 7) (catalog no. #3801), anti‐AGT (angiotensinogen) (catalog no. #79299), anti‐PAI‐1 (plasminogen activator inhibitor‐1) (catalog no. #11907), anti‐Axin2 (catalog no. #2151), anti‐Cyclin D1 (catalog no. 2978) and anti‐Snail (catalog no. #3879) were from Cell Signaling Technology (Beverley, MA, USA). Lipofectamine 2000 (catalog no. 11668019) was from Invitrogen (Carlsbad, CA). Recombinant IFN‐γ (interferon γ) (catalog no. PHC4033) was obtained from ThemoFisher Scientific (Waltham, MA, USA).

### 2.2 Podocyte culture and treatments

The conditionally immortalized mouse podocyte cell line was kindly provided by Prof. Chuan‐Ming Hao (Department of Neurology, Huashan Hospital, Fudan University). The cell culture medium was composed of RPMI‐1640 medium, 10% fetal bovine serum (FBS) and recombinant IFN‐γ. Cells were cultured at 33°C in cell incubator. To induce differentiation, recombinant IFN‐γ was removed from culture medium and podocytes were grown at 37°C.^22^ Cells were transfected with the plasmid expressing Wnt1 (Addgene, catalog no. #35905) 23 or β‐catenin S33Y (Addgene, catalog no. #19286) 24 using Lipofectamine. At 12 hour‐post transfection, cells were starved in serum‐free medium for 6 hours and then cells were treated with 1 μg/mL of ADR in the presence or absence of salidroside (100 μmol/L) for additional 12 hours. To examine effects of salidroside on β‐catenin activity, cells were transfected with the plasmid bearing Wnt1 or β‐catenin S33Y. At 6 hour‐post transfection, salidroside (100 μmol/L) was added and cells were cultured for additional 18 hours. At the end of treatments, cells were harvested for further analysis.

### 2.3 Animal models

All experiments involving animals were approved by the Institutional Animal Care and Use Committees of Nantong University (Approval ID: SYXK [SU] 2017‐0046). The mouse model of podocyte injury and proteinuria was accomplished by injection of ADR via tail vein at the dosage of 10 mg/kg body wt.^25^ After 1 week of adaptive feeding, 6‐week‐old male BALB/c mice (20‐22 g) were randomly assigned to the following three treatment groups: (1) control (n = 5); (2) ADR‐treated mice (n = 5); and (3) ADR‐treated mice with Sal (n = 5). Salidroside was dissolved in drinking water (0.6 mg/mL) and given to mice after ADR injection. According to the average daily water consumption, the current treating regimen leads to a dosage of 100 mg kg^−1^ d^−1^ salidroside. Urine and kidney tissues were collected at 5 weeks for further analysis.

### 2.4 Urine creatinine and albumin detection

Urine creatinine, blood urea nitrogen and albumin were analysed by mouse ELISA (enzyme linked immunosorbent assay) kits (Sigma‐Aldrich) according to the manufacturer's instructions.

### 2.5 Morphometry and histology

Kidney samples were fixed in 4% paraformaldehyde at 4°C for 24 hours. The paraffin‐embedded kidney tissues were cut into 3 μm thickness and stained with Masson‐trichrome and Periodic acid‐Schiff (PAS) staining for histological analysis. Quantitation of the fibrotic area was carried out by computer‐aided morphometric analysis software (Image‐pro plus 6).^26^ Mesangial matrix fraction was analysed by the method described elsewhere.^27^

### 2.6 Immunofluorescence and immunohistochemistry

Heat‐mediated antigen retrieval was performed using citrate buffer. Slides were then incubated with the primary antibodies (anti‐nephrin and anti‐podocin) overnight at 4°C. Finally, the colour reaction was initiated by adding 3, 3^’^‐diaminobenzidine tetrahydrochloride substrate. For immunofluorescence, slides were blocked with 10% bovine serum for 30 minutes and immunostained with the primary antibodies.

### 2.7 Transmission electron microscope

The preparation of kidney samples for transmission electron microscope was described elsewhere.^28^, ^29^ Briefly, the samples were fixed in cacodylate buffer (0.1 mol/L, pH 7.4) containing 2.5% paraformaldehyde and 2.5% glutaraldehyde. After fixation, samples were placed in 1% osmium tetraoxide at 4°C for 1 hour. Then samples were subjected for dehydration by graded alcohol (50, 70, 90 and 100%). Samples were oriented longitudinally and embedded in Epon 812. Embedded samples were cut into 70 nm thickness and the resulted sections were contrasted in lead citrate and uranyl acetate. Finally, sections were examined with a transmission electron microscope at 80 kV (JEO Ltd., Tokyo, Japan).

### 2.8 Tissue and cell protein extraction

Tissues or cells were homogenized with a homogenizer (Polytron, PT2100) in ice‐cold lysis buffer, which was composed of 25 mmol/L Tris‐HCl, pH 7.4; 50 mmol/L Na_4_P_2_O_7_; 100 mmol/L NaF; 10 mmol/L Na_3_VO_4_; 10 mmol/L EDTA; 10 mmol/L EGTA; 10 μg/mL Leupeptin; 1% NP‐40; 10 μg/mL Aprotinin; 20 nmol/L Okadaic acid and 2 mmol/L PMSF (phenylmethanesulfonyl fluoride). Then lysates were subjected to centrifugation at 13 800 *g* at 4°C for 20 minutes. The supernatant was collected and protein concentration was analysed by a Protein Assay Kit (Bio‐Rad). Samples were boiled for 5 minutes at 100°C in 1X Laemmli buffer.

### 2.9 Nuclear and cytoplasmic protein isolation

The cytoplasmic and nuclear protein extraction was prepared by a commercial kit (Thermo Scientific, catalog no. 78835) according to the manufacturer's instruction. In brief, tissues or cells were washed twice with cold PBS and transferred into Eppendorf tubes for centrifugation at 4°C for 5 minutes at 500 *g*. The supernatant was discarded and leaving pellet as dry as possible. Tissue homogenization was performed using tissue grinders in the 400 μL of CER I and homogenate was subjected to vortex for 15 seconds to fully suspend the pellet and placed on ice for 10 minutes, then added cold 22 μL CER II to the tubes. Samples were subjected to vortex for 5 seconds, then incubated on ice for 1 minute and centrifuged at 16 000 *g* for 5 minutes. The supernatant was assigned as cytoplasmic protein fraction and immediately transferred to a clean ice‐cooled tube. Suspend the insoluble (pellet) fraction in cold 200 μL NER and thoroughly vortex 15 seconds. The vortex was repeated three more times and the mixture was subjected to centrifugation. The resulting supernatant was considered as nuclear protein fraction.

### 2.10 RNA extraction and quantitative real time PCR (qRT‐PCR)

The procedures for RNA extraction and quantitative real time PCR were described previously.^29^ Trizol reagent (Invitrogen) was used to total RNA extraction from animal tissues. The resulting RNA was used as templates for cDNA synthesis. The mRNA expression was analysed with SYBR Green Supermix using iQ5 Multicolor Real‐Time PCR Detection System (Bio‐Rad). The primer sequences were as follows: α‐SMA, 5^’^‐GAGGCACCACTGAACCCTAA‐3^’^ (forward) and 5^’^‐CATCTCCAGAGTCCAGCACA‐3^’ ^(reverse); collagen Ⅰ, 5^’^‐GTAACTTCGTGCCTAGCAACA‐3^’^ (forward) and 5^’^‐CCTTTGTCAGAATACTGAGCAGC‐3^’ ^(reverse); Axin2, 5’‐TGACTCTCCTTCCAGATCCCA‐3’ (forward) and 5’‐TGCCCACACTAGGCTGACA‐3’ (reverse); Cyclin D1, 5’‐GCGTACCCTGACACCAATCTC‐3’ (forward) and 5’‐CTCCTCTTCGCACTTCTGCTC‐3’ (reverse).

### 2.11 Western blot analysis

Western blot analysis was described previously.^30^ Protein samples were resolved by SDS‐PAGE (sodium dodecyl sulphate‐polyacrylamide gel electrophoresis) and transferred to polyvinylidene fluoride (PVDF) membrane. Membranes were incubated in 10% blocking reagent (Roche) for 1 hour at room temperature and incubated with specific primary antibodies overnight at 4°C. Membranes were washed three times in TBST (Tris‐Buffered Saline and Tween 20) and then incubated with the secondary antibody at room temperature for 1 hour. After incubation, membranes were washed three times in TBST. Finally, membranes were developed using a chemiluminescence assay system (Roche) and signals were exposed to films. Protein levels were quantified by Image J program.

### 2.12 Statistical analysis

Data are expressed as the mean ± standard error of the mean (SEM). Statistical analysis was performed using one‐way analysis of variance followed by the Dunnett's *post hoc* test with SPSS 18.0 software. A value of *P* < 0.05 was the threshold used for significance.

## 3 RESULTS

### 3.1 Salidroside reduces proteinuria in ADR nephropathy

**Figure 1:**
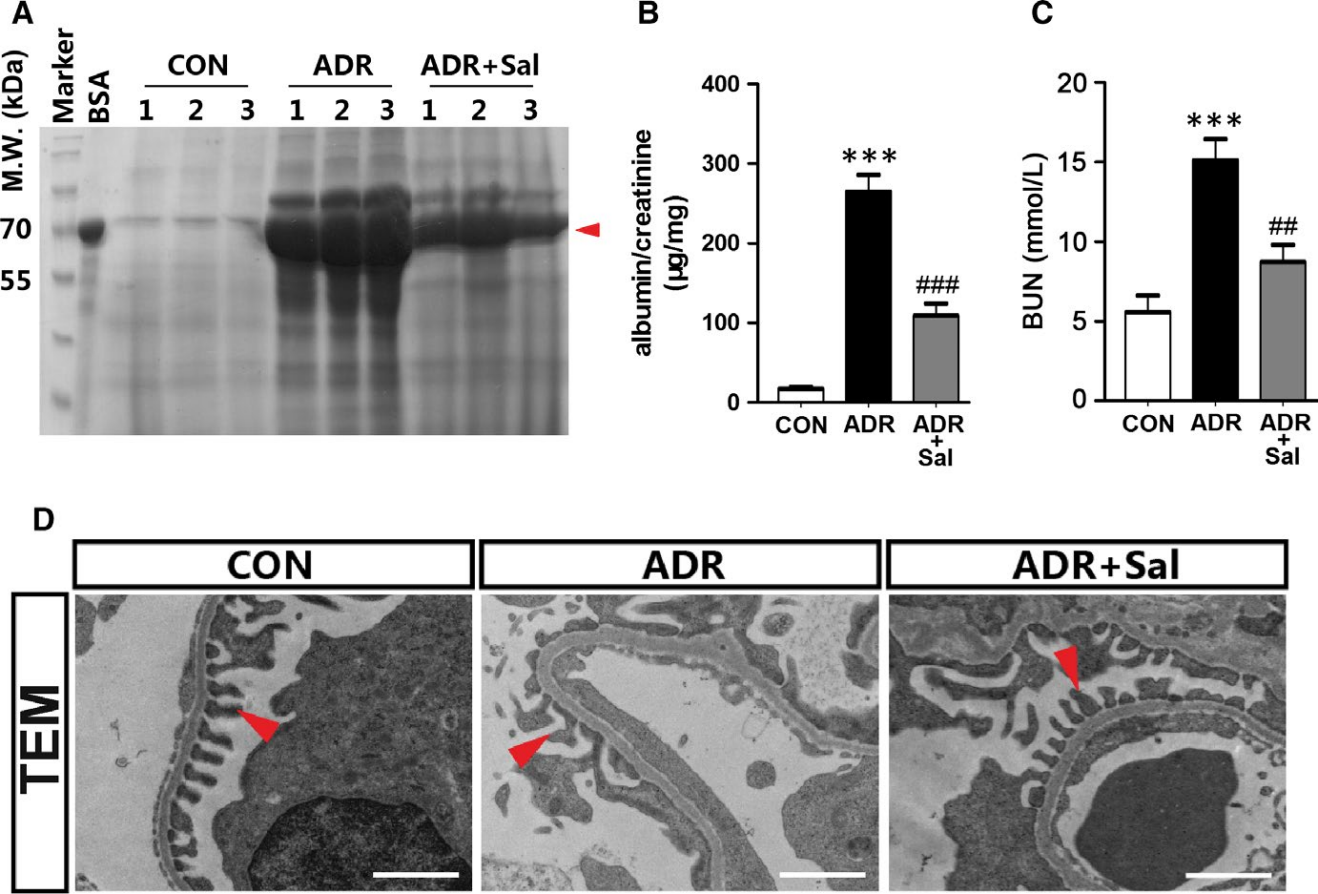
Salidroside administration reduces proteinuria in ADR nephropathy. (A) SDS‐PAGE analysis showing the abundance of urinary proteins. Red arrow indicates the size of urinary proteins. (B) Urinary albumin levels. (C) Blood urea nitrogen (BUN) concentrations. (D) TEM analysis showing podocyte foot processes integrity. Arrowheads indicate secondary foot process and SD. Scale bar = 1 µm. 24‐hour urine samples were used for detecting urinary proteins and urinary albumin levels. The results are the means ± SEM of three independent experiments. ***P < 0.001 vs CON; ^##^ *P* < 0.01 and ^###^ *P* < 0.001 vs ADR alone. n = 5. ADR, adriamycin; CON, control; Sal, salidroside; TEM, transmission electron microscope

Adriamycin (ADR)‐induced nephropathy is a widely used and well characterized model of initial podocyte injury, albuminuria, hereafter renal inflammation and fibrosis in mice.^31^ To examine the potential roles of salidroside on nephropathy, ADR‐induced nephropathy model was employed. As shown in Figure 1A, the urinary albumin levels were significantly increased by ADR. The application of salidroside markedly mitigated proteinuria induced by ADR. To further confirm these results, we measured urinary albumin and blood urea nigtrogen (BUN) concentrations. The results showed that salidroside treatment significantly attenuated the increased levels of urinary albumin and BUN in ADR‐treated animals (Figure 1B,C). Furthermore, the ultrastructural analysis showed that ADR treatment induced more severe foot process effacement. The application of salidroside greatly alleviated these detrimental effects (Figure 1D). These data clearly indicate that salidroside treatment preserves podocyte integrity and alleviates ADR‐induced albuminuria.

### 3.2 Salidroside administration ameliorates kidney injury in ADR nephropathy

**Figure 2:**
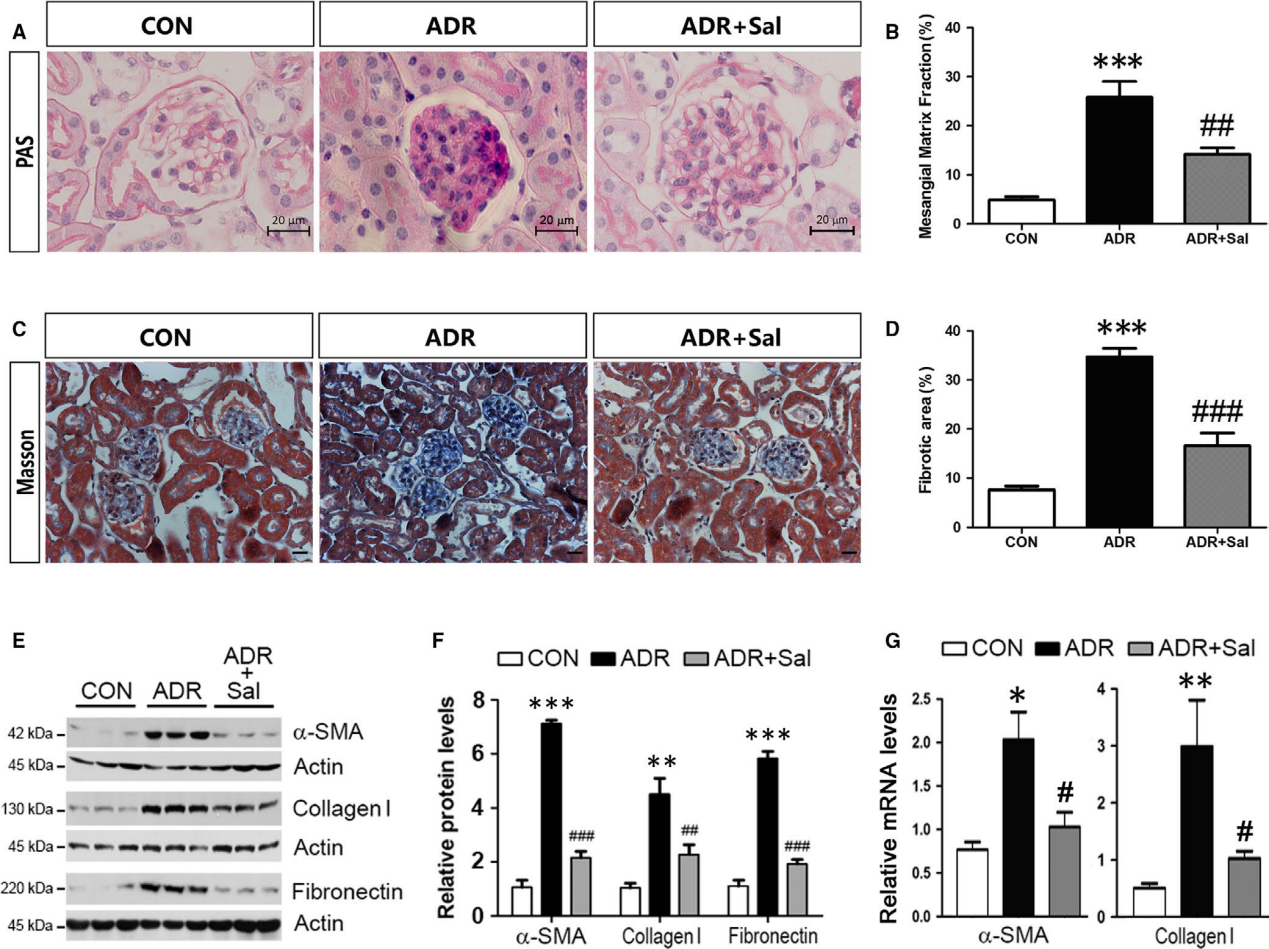
Salidroside administration ameliorates kidney injury in ADR nephropathy. (A) PAS staining. Representative micrographs were shown. Scale bar = 20 µm. (B) Morphometric analysis of PAS stained kidney sections. (C) Masson‐trichrome staining. Representative micrographs were shown. Scale bar = 20 µm. (D) Morphometric analysis of Masson‐trichrome stained kidney sections. (E) Western blot analyses showing the protein levels of α‐SMA, collagen I and fibronectin. (F) Quantitative determination for α‐SMA, collagen I and fibronectin protein levels. (G) The mRNA levels of α‐SMA and collagen I are shown. The results are the means ± SEM of three independent experiments. *P < 0.05, **P < 0.01 and ***P < 0.001 vs CON; ^#^ *P* < 0.05, ^##^ *P* < 0.01 and ^###^ *P* < 0.001 vs ADR alone. n = 5. ADR, adriamycin; CON, control; Sal, salidroside

Next, we examined whether salidroside ameliorates renal injury in ADR‐treated mice. According to PAS staining, ADR treatment induced a significant increase in glomerular extracellular matrix (ECM) deposition, which was reduced by salidroside (Figure 2A,B). Masson‐trichrome staining showed that the fibrotic lesion was occurred in ADR‐treated animals, which was attenuated in the presence of salidroside (Figure 2C,D). The enhanced expression of α‐smooth muscle actin (α‐SMA) is a promising marker of myofibroblasts.^32^ Therefore, we measured the levels of α‐SMA and the results showed that the expression of α‐SMA was dramatically increased by ADR; and this increase was largely counteracted by salidroside (Figure 2E,F). Additionally, we also analysed expressions of fibronectin and collagen I. Similarly, salidroside treatment significantly attenuated the ADR‐induced increases in fibronectin and collagen I (Figure 2E,F). These changes as aforementioned were further confirmed by the changes in the mRNA levels of α‐SMA and collagen I (Figure 2G). These results further ascertain that salidroside treatment is an effective intervention for preventing ADR‐induced nephropathy in mice.

### 3.3 Salidroside preserves podocin and nephrin expression

**Figure 3:**
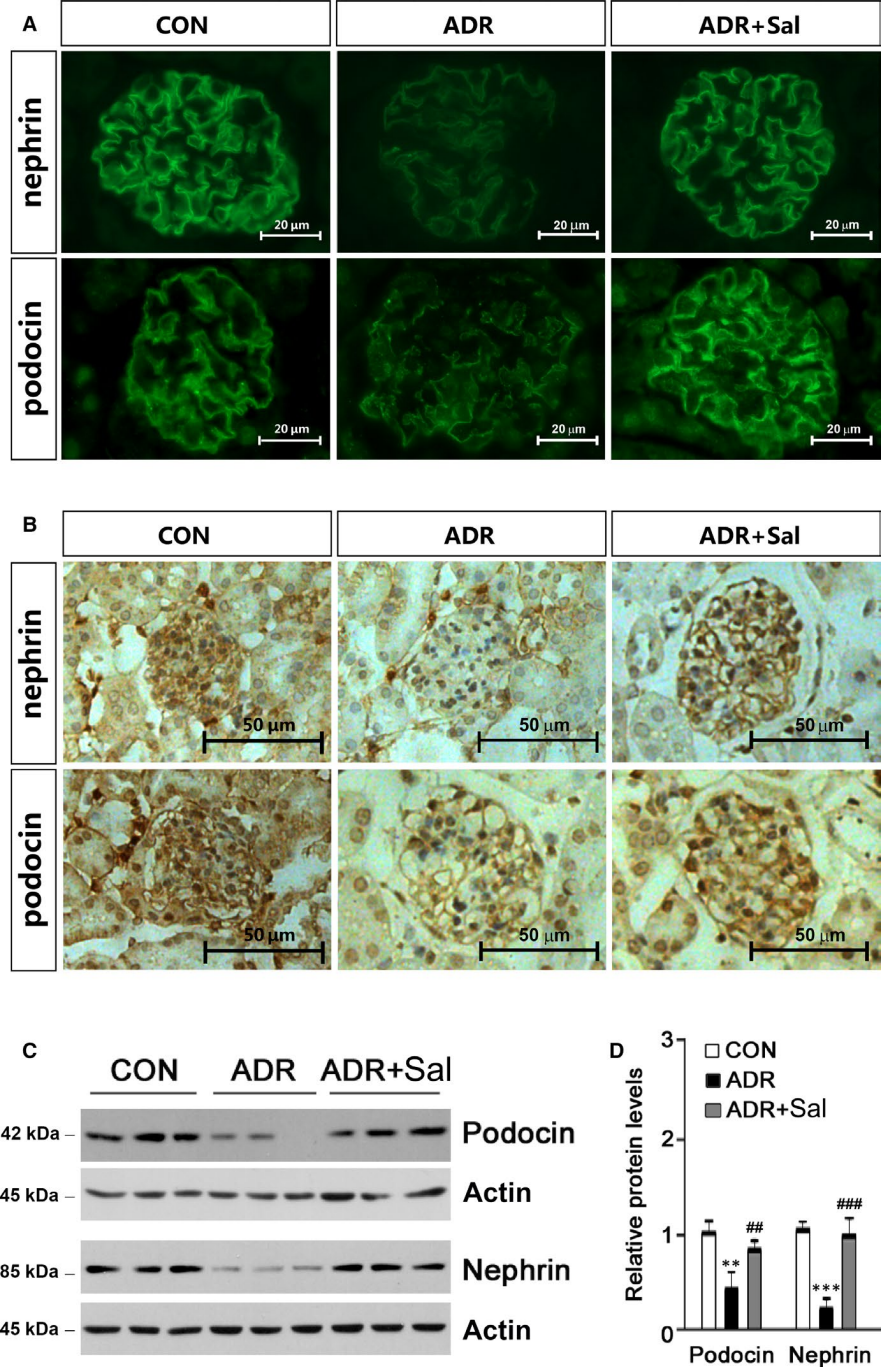
Salidroside preserves nephrin and podocin expressions and prevents podocyte injury in ADR nephropathy. (A) Immunofluorescence analysis showing the abundance and distribution of nephrin and podocin. Representative micrographs were given. Scale bar = 20 µm. (B) Immunohistochemical analysis showing nephrin and podocin expressions. Representative micrographs were given. Scale bar = 50 µm. (C) Expressions of nephrin and podocin were analysed by Western blot. Glomerular lysates from different groups of mice were immunoblotted with specific antibodies against nephrin and podocin respectively. Actin was used as a loading control. (D) Quantitative determination of the relative abundances of nephrin and podocin. The results are the means ± SEM of three independent experiments. **P < 0.01 and ***P < 0.001 vs CON; ^##^ *P* < 0.01 and ^###^ *P* < 0.001 vs ADR alone. n = 5. ADR, adriamycin; CON, control; Sal, salidroside

ADR treatment has been shown to induce podocyte injury by reducing podocin and nephrin expressions, which are slit diaphragm (SD)‐associated proteins.^33^ Thus, the effects of salidroside on nephrin and podocin expressions were examined. As shown in Figure 3A, the expressions of nephrin and podocin were substantially decreased in ADR‐treated animals; the application of salidroside restored the expressions of nepherin and podocin. The immunohistochemical analysis also showed similar changes in nephrin and podocin expressions induced by ADR and salidroside (Figure 3B). To further confirm these findings, the protein levels of podocin and nephrin were analysed by Western blot. The results showed that both podocin and nephrin were reduced in ADR‐treated animals; the treatment of salidroside greatly prevented these declines (Figure 3C,D).

### 3.4 Salidroside blocks renal β‐catenin activation induced by ADR

**Figure 4:**
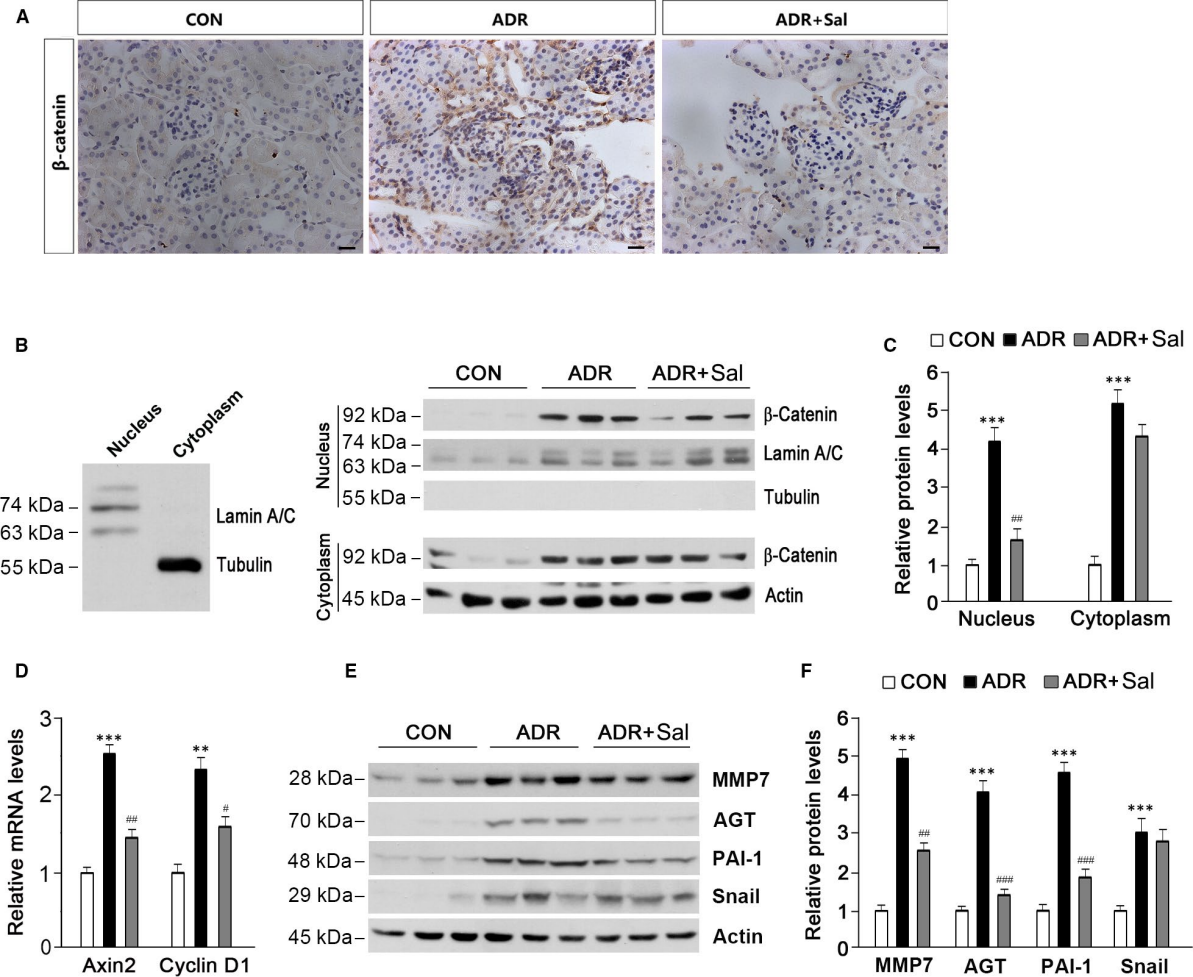
Salidroside administration blocks renal β‐catenin activation induced by ADR. (A) Immunohistochemical analysis showing β‐catenin protein expression and localization in the kidney. Representative micrographs were shown. Scale bar = 20 µm. (B) Western blot analysis showing renal β‐catenin protein abundance in the nucleus and cytoplasm. Nucleus and cytoplasm lysates were distinguished using antibodies against Lamin A/C and Tubulin respectively (left panel). Lamin A/C and Actin were used as loading controls. (C) Quantitative analysis of β‐catenin protein levels as shown in (B). (D) Axin2 and Cyclin D1 expressions were examined. (E) Western blot analysis showing the effects of salidroside on β‐catenin downstream target gene expression. (F) Quantitative analysis of protein levels as shown in (E). Lamin A/C and Actin were used as loading controls. The results are the means ± SEM of three independent experiments. **P < 0.01; ***P < 0.001 vs CON; ^#^ *P* < 0.05; ^##^ *P* < 0.01, ^###^ *P* < 0.001 vs ADR alone. n = 5. ADR, adriamycin; AGT, angiotensinogen; CON, control; MMP7, matrix metalloproteinase 7; PAI‐1, plasminogen activator inhibitor‐1; Sal, salidroside

Previous reports have shown that ADR nephropathy is often associated with Wnt signalling pathway activation.^2^, ^5^, ^34^ As β‐catenin is the principal factor of the canonical Wnt signalling pathway, we therefore examined whether salidroside alters β‐catenin activity. Immunohistochemical analysis showed that β‐catenin expression was markedly increased by ADR, which was attenuated in the presence of salidroside (Figure 4A). To examine subcellular localization of β‐catenin, nuclear and cytoplasmic protein fractions were prepared for Western blot analysis. The purity of nuclear and cytoplasmic protein fractions was confirmed by their respective markers (Figure 4B, left panel). The results showed that ADR treatment increased β‐catenin expression in the nucleus and cytoplasm; the application of salidroside greatly prevented the accumulation of β‐catenin in the nucleus (Figure 4B,C). No signals for α‐Tubulin were observed in the nuclear protein fraction (Figure 4B). Axin2 and Cyclin D1 are the two main downstream effectors of β‐catenin. As shown in Figure 4D, ADR induced significant increases in Axin2 and Cyclin D1 expressions; salidroside could repress these increases. To further confirm the changes in β‐catenin, several downstream target genes of β‐catenin were analysed. MMP7, AGT and PAI‐1 were robustly stimulated by ADR; however, these stimulations were markedly decreased in the presence of salidroside (Figure 4E,F). As for Snail, it was not altered by salidroside in ADR‐treated animals (Figure 4E,F). These results suggest that the beneficial roles of salidroside on ADR nephropathy may rely on its inhibitory effect on β‐catenin nuclear translocation.

### 3.5 β‐catenin activation abolishes the protective roles of salidroside in ADR‐injured podocytes

**Figure 5:**
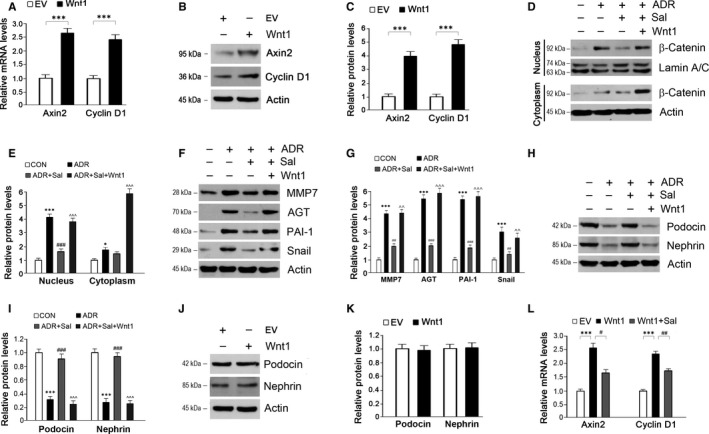
Activation of β‐catenin by Wnt1 abolishes the protective effects of salidroside against ADR injury in podocytes. (A‐B) The transfection of Wnt1 in cultured podocytes successfully activates β‐catenin activity. (C) Quantitative analysis of Axin2 and Cyclin D1 as shown in (B). (D) The repression of salidroside on β‐catenin activity was counteracted by Wnt1 in ADR‐treated podocytes. (E) Quantitative analysis of Western blots as shown in (D). (F) The inhibition of β‐catenin downstream target gene expressions by salidroside was re‐activated by Wnt1 in ADR‐treated podocytes. (G) Quantitative analysis of Western blots as shown in (F). (H) Activation of β‐catenin by Wnt1 reverses the effects of salidroside on podocin and nephrin expressions in ADR‐injured podocytes. (I) Quantitative analysis of Western blots as shown in (H). (J) Wnt1 has no effects on podocin and nephrin expressions. (K) Quantitative analysis of Western blots as shown in (J). (L) Effects of salidroside on Axin2 and Cyclin D1 expressions in podocytes transfected with the plasmid bearing Wnt1. Lamin A/C and Actin were used as loading controls. The results are the means ± SEM of three independent experiments. EV, empty vector; CON, control; ADR, adriamycin; Sal, salidroside; MMP7, matrix metalloproteinase 7; AGT, angiotensinogen; PAI‐1, plasminogen activator inhibitor‐1. *P < 0.05 and ***P < 0.001 vs CON; ^#^ *P* < 0.05; ^##^ *P* < 0.01 and ^###^ *P* < 0.001 vs ADR; ^^*P* < 0.01 and ^^^*P* < 0.001 vs ADR + Sal

To confirm whether the observed protective effects of salidroside against ADR nephropathy are dependent upon β‐catenin inactivation, we activated β‐catenin pathway and examined the effects of salidroside in ADR‐injured podocytes. Wnt1 is an upstream inducer of β‐catenin signalling pathway 23; and β‐catenin S33Y is a constitutively activated form of β‐catenin.^24^ Hence, these two plasmids were employed to activate β‐catenin pathway. First, we examined whether Wnt1 activates β‐catenin activity in cultured podocytes. Our results showed that the transfection of Wnt1 markedly increased expressions of Axin2 and Cyclin D1 (Figure 5A‐C). Next, we sought to investigate whether Wnt1 affects the effects of salidroside on podocyte function in the presence of ADR. As shown in Figure 5D, ADR treatment induced a marked increase in β‐catenin accumulation in the nucleus, whereas salidroside markedly prevented this accumulation. As expected, overexpression of Wnt1 abolished the inhibitory effect of salidroside on β‐catenin nuclear translocation (Figure 5D,E). As for β‐catenin downstream target genes, their expressions were activated by ADR and the presence of salidroside largely blocked these activations (Figure 5F,G). Similar to the results observed in vivo, ADR treatment reduced podocin and nephrin expressions, and salidroside could prevent these declines (Figure 5H,I). Wnt1‐mediated β‐catenin activation almost completely abolished the effects of salidroside on podocin and nephrin expressions in cultured podocytes (Figure 5H,I). To exclude the possibility that Wnt1 alone induced reductions in podocin and nephrin expressions, we measured the protein levels of podocin and nephrin and the results showed that the transfection of Wnt1 had no effect on podocin and nephrin in cultured podocytes (Figure 5J,K). Furthermore, salidroside repressed Axin2 and Cyclin D1 expressions in cells transfected with the plasmid bearing Wnt1 (Figure 5L).

**Figure 6:**
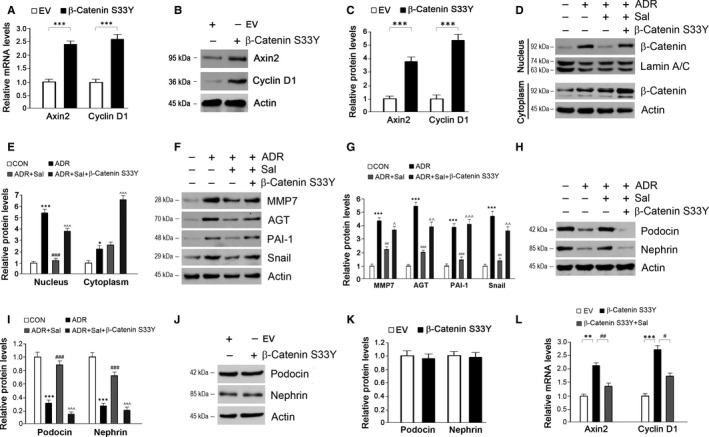
Activation of β‐catenin by β‐catenin S33Y diminishes the protective roles of salidroside against ADR injury in podocytes. (A‐B) The transfection of β‐catenin S33Y in cultured podocytes successfully activates β‐catenin activity. (C) Quantitative analysis of Axin2 and Cyclin D1 as shown in (B). (D) The repression of salidroside on β‐catenin activity was counteracted by β‐catenin S33Y in ADR‐treated podocytes. (E) Quantitative analysis of Western blots as shown in (D). (F) The inhibition of β‐catenin downstream target gene expressions by salidroside was re‐activated by β‐catenin S33Y in ADR‐treated podocytes. (G) Quantitative analysis of Western blots as shown in (F). (H) Activation of β‐catenin by β‐catenin S33Y reverses the effects of salidroside on podocin and nephrin expressions in ADR‐injured podocytes. (I) Quantitative analysis of Western blots as shown in (H). (J) β‐catenin S33Y has no effects on podocin and nephrin expressions. (K) Quantitative analysis of Western blots as shown in (J). (L) Effects of salidroside on Axin2 and Cyclin D1 expressions in podocytes transfected with the plasmid bearing β‐catenin S33Y. Lamin A/C and Actin were used as loading controls. The results are the means ± SEM of three independent experiments. EV, empty vector; CON, control; ADR, adriamycin; Sal, salidroside; MMP7, matrix metalloproteinase 7; AGT, angiotensinogen; PAI‐1, plasminogen activator inhibitor‐1. *P < 0.05; **P < 0.01 and ***P < 0.001 vs CON; ^#^ *P* < 0.05; ^##^ *P* < 0.01 and ^###^ *P* < 0.001 vs ADR; ^*P* < 0.05; ^^*P* < 0.01 and ^^^*P* < 0.001 vs ADR + Sal

To further confirm the above findings, we activated β‐catenin by the transfection of β‐catenin S33Y. As well as Wnt1, β‐catenin S33Y stimulated Axin2 and Cyclin D1 expressions in cultured podocytes (Figure 6A‐C). The nuclear translocation of β‐catenin was reinforced by β‐catenin S33Y in the presence of salidroside and ADR (Figure 6D,E). The inhibitory effects of salidroside on the downstream gene expressions were counteracted by β‐catenin S33Y (Figure 6F,G). The improvements of salidroside on podocin and nephrin were diminished by β‐catenin S33Y (Figure 6H,I). No significant changes in podocin and nephrin were observed in the cells transfected with β‐catenin S33Y (Figure 6J,K). Salidroside inhibited Axin2 and Cyclin D1 expressions induced by β‐catenin S33Y (Figure 6L). These results clearly indicate that the protective roles of salidroside against ADR injury in podocytes are dependent on its repression of β‐catenin activity.

## 4 DISCUSSION

In this study, we have shown that salidroside attenuated ADR nephropathy in mice. For example, ADR‐induced proteinuria and renal fibrosis were reduced by the application of salidroside; meanwhile, the decreases in nephrin and podocin expressions were counteracted by salidroside. Our data suggest that salidroside might be used as a drug or pro‐drug for preventing proteinuric renal diseases.

Chronic kidney disorder (CKD) is a public health problem worldwide, because of the high burden, such as decreased productivity, related adverse outcomes and associated costs.^35^ Increasing evidence suggests that proteinuria is a strong indicator and considered as one of the risk factors for CKD progression.^36^ In the present study, our data showed that ADR‐induced proteinuria was markedly attenuated by salidroside. In line with our findings, Wu et al also observed that salidroside alleviates diabetic albuminuria by down‐regulation of Cav‐1 phosphorylation and inhibition of albumin transcytosis across glomerular endothelial cells.^16^ In addition, other traditional Chinese medicines also have similar effects on reducing proteinuria. For instance, An et al observed that hyperoside, a flavone glycoside with anti‐inflammatory, anti‐cancer and anti‐oxidan properties, prevents proteinuria in diabetic nephropathy.^37^ Furthermore, Bai et al found that Huayu Tongluo herbs inhibits the production of proteinuria in diabetic rats.^38^

Mounting evidence indicates that podocyte dysfunction plays a key role for proteinuria. Podocyte injury is manifested in a variety of forms, which include the deletion or mutation of the slit diaphragm‐associated proteins (eg, podocin and nephrin).^39^ In the current study, we observed that ADR‐induced reductions in podocin and nephrin were restored partially in the presence of salidroside. Furthermore, the beneficial roles of salidroside against ADR nephropathy are likely mediated by its inhibition on β‐catenin activity. Wnt/β‐catenin signalling pathway is closely related with various developmental events.^40^ It plays an essential role in tissue homeostasis, organogenesis and pathogenesis of many human disorders.^41^ Previous study has shown that Wnt/β‐catenin signalling has a key effect on podocyte injury and dysfunction, thereby progressing to proteinuria.^42^ Wnt/β‐catenin pathway is activated in a wide variety of kidney disorders, including diabetic nephropathy,^43^ focal and segmental glomerulosclerosis 44 and Adriamycin nephropathy.^45^ In the present work, we observed that β‐catenin accumulates both in the cytoplasm and nucleus after ADR administration. In the presence of salidroside, however, the distribution of β‐catenin in the nucleus was decreased. Similarly, it has been shown that, by inhibiting Wnt/β‐catenin, paricalcitol improves podocyte function, alleviates kidney injury and proteinuria in ADR nephropathy.^2^ Most recently, one study has shown that salidroside activates β‐catenin pathway in the substantia nigra of rats,^46^ which is contrary to our result that salidroside inhibits β‐catenin activity. This discrepancy is probably due to the differences in the dosages of salidroside (250 mg kg^−1^ d^−1^ vs 100 mg/kd/day), the species (rat vs mouse), or the target tissues (the substantia nigra vs the kidney).

Recently, Renin‐Angiotensin system (RAS) was characterized as a substrate gene of Wnt/β‐catenin in ADR nephropathy.^45^ Inhibition of RAS greatly attenuates ADR‐induced kidney injury and proteinuria.^45^ In the present study, we also found that AGT, a member of RAS, was stimulated by ADR and down‐regulated in the presence of salidroside. To further ascertain the significance of β‐catenin inhibition on the protective roles of salidroside in ADR nephropathy, we activated β‐catenin in cultured podocytes by transfection of the plasmid expressing Wnt1 or β‐catenin S33Y. Indeed, the activation of β‐catenin almost completely abolished the effects of salidroside in ADR‐injured podocytes. In addition, we noticed that salidroside treatment has no effect on Snail expression in ADR‐treated animals; however, in cultured podocytes, salidroside greatly prevents the increase in Snail induced by ADR. This discrepancy may be due to the different experimental models, that is, in vitro and in vivo. Another issue that caused our attention is, salidroside has no effect on β‐catenin nuclear translocation in the cells transfected with Wnt1. The underlying molecular mechanisms are still not clear. One possible reason is that, Wnt1 binds to its receptor and results in the receptor autophosphorylation and activation, which stimulates recruitment of the Axin complex to the receptors. These events lead to inhibition of Axin‐mediated β‐catenin phosphorylation and thereby to the stabilization of β‐catenin. This Wnt1‐induced interaction between Axin complex and the receptors may affect the effects of salidroside on Axin complex. Therefore, salidroside fails to induce β‐catenin nuclear translocation in the presence of Wnt1.

In conclusion, our data indicate that salidroside mitigates ADR induced podocyte dysfunction, reduces proteinuria and kidney injury; and these beneficial roles may rely on inhibition of Wnt/β‐catenin signalling pathway. These novel findings may provide new insights for developing therapeutic modalities based on salidroside for preventing proteinuric kidney diseases.

## CONFLICT OF INTEREST

The authors declare that they have no conflict of interest.
